# Adult Hippocampal Neurogenesis and Affective Disorders: New Neurons for Psychic Well-Being

**DOI:** 10.3389/fnins.2021.594448

**Published:** 2021-06-16

**Authors:** Walace Gomes-Leal

**Affiliations:** ^1^Post-Graduation Program in Health Sciences, Institute of Collective Health, Federal University of Western Pará, Santarém, Brazil; ^2^Post-Graduation Program in Pharmacology and Biochemistry, Institute of Biological Sciences, Federal University of Pará, Belém, Brazil

**Keywords:** hippocampus, adult neurogenesis, pattern separation, cognitive flexibility, anxiety, depression, brain evolution

## Abstract

A paradigm shift in neuroscience was the discovery that new neurons are constantly produced in the adult mammalian brain of several species, including Homo sapiens. These new-born cells are formed in some main neurogenic niches, including the subventricular zone (SVZ) at the margin of the lateral ventricle and subgranular zone (SGZ) in the hippocampal dentate gyrus (DG). In the DG, neuroblasts derive from SGZ progenitors and migrate to the hippocampal granular layer becoming adult granule cells, which are integrated into functional adult circuits. It has been confirmed that adult hippocampal neurogenesis (AHN) is a long-lasting phenomenon in the human brain. The functions of hippocampal new-born cells are not fully established. Experimental studies suggest that they have unique electrophysiological properties, including hyperexcitability, which enable them to regulate adult granule cells. Their specific function depends on the anatomical hippocampal location along the hippocampal dorsal-ventral axis. Dorsal hippocampus plays a more defined role on spatial learning and contextual information, while the ventral hippocampus is more related to emotional behavior, stress resilience and social interaction. Several reports suggest a role for AHN in pattern separation, cognitive flexibility, forgetting and reversal learning. It has been proposed that deficits in AHN might impair normal DG function, including pattern separation and cognitive flexibility, which could play a role on the etiology of affective disorders, such as depression, anxiety and post-traumatic stress disorder (PTSD). In this paper, we review recent scientific evidence suggesting that impairment of AHN may underlie the pathophysiology of affective disorders even in humans and that neurogenesis-inspired therapies may be a promising approach to reduce symptoms of affective disorders in humans.

## Introduction

One of the most remarkable discoveries in neuroscience was that new neurons are constantly produced in the adult brain of several mammalian species (Altman and Das, 1965; Gage, 2019; Lima and Gomes-Leal, 2019), including Homo sapiens (Eriksson et al., 1998; Spalding et al., 2013; Boldrini et al., 2018; Moreno-Jiménez et al., 2019). This was a 30-year battle against skepticism of scientific community (Lima and Gomes-Leal, 2019), since the pioneering work of Joseph Altman and his colleagues in the 1960s suggesting the existence of neurogenesis in the adult brain (Altman and Das, 1965). In the 1990s, the availability of modern techniques for the unequivocal labeling of adult-born neurons established as irrefutable that new neurons are produced into specific regions of central nervous system (CNS), including subventricular zone (SVZ) (Doetsch et al., 1997, 1999) at the margin of the lateral ventricle and subgranular zone (SGZ) in the dentate gyrus (DG) of hippocampus (Cameron and Gould, 1994; Kuhn et al., 1996). Recently, striatum has also been reported as a neurogenic niche in both rodents and humans (Ernst et al., 2014; Ernst and Frisén, 2015). Striatal neurogenesis was reported to be higher in humans than in rodents due to increased complexity and peculiarities of human cognition (Ernst and Frisén, 2015).

Several scientific evidences point to an important role of adult hippocampal neurogenesis (AHN) on the normal DG function. These functions include pattern separation, cognitive flexibility, forgetting and reversal learning (Anacker and Hen, 2017). Based on animal experiments it has been proposed that impairment of AHN as a consequence of chronic stress might impair the normal physiology of adult hippocampus, which could underlie the etiology of affective disorders, including depression, anxiety and post-traumatic stress disorder (PTSD) (Anacker and Hen, 2017; Toda et al., 2019; Murthy and Gould, 2020). This raises the interesting possibility that neurogenesis-inspired therapies, including aerobic exercise and meditation, may become a reality and an important tool in psychiatry and psychology (Shors et al., 2014; Millon and Shors, 2019; Lavadera et al., 2020).

In this paper, I review recent scientific evidence suggesting that impairment of AHN may underlie the pathophysiology of affective disorders, even in humans. I emphasize that modulation of AHN by pharmacological or non-pharmacological therapies (including aerobic exercise) may become a pivotal approach to diminish mental suffering in people afflicted by affective disorders. I also discuss the importance of physical activity for mental health as consequence of our evolutive history and a new hypothesis to explain the human correlate of enriched environment.

## Functions of Adult Hippocampal Neurogenesis

While the existence of adult neurogenesis in the human SVZ is yet debatable (Curtis et al., 2007; Sanai et al., 2011), AHN has been firmly established in the human brain (Eriksson et al., 1998; Spalding et al., 2013; Boldrini et al., 2018; Moreno-Jiménez et al., 2019), despite this has been recently disputed (Sorrells et al., 2018). The function of these adult-born cells remains under investigation (Gage, 2019; Lima and Gomes-Leal, 2019). Nevertheless, studies in rodents suggest that new-born cells play an important role on the normal DG function, due to their peculiar electrophysiological properties, including hyperexcitability (Anacker and Hen, 2017; Vicidomini et al., 2020). In the mouse DG, new neurons differentially modulate different inputs from both lateral and medial entorhinal cortex, which have profound influence on DG normal physiology (Luna et al., 2019). Proposed functions for hippocampal adult-born neurons, include hippocampus-dependent learning tasks in which temporal association is needed (Aimone et al., 2006; Rangel et al., 2014), spatial contextual navigation in new environments (Kempermann et al., 1997), pattern separation (Nakashiba et al., 2012; Niibori et al., 2012), forgetting (Akers et al., 2014), memory clearance (Epp et al., 2016) and cognitive flexibility (Burghardt et al., 2012; Park et al., 2015). These functions are dependent on the interplay between adult-born hippocampal cells and old granule cells and their synaptic connections with other hippocampal regions, including CA3 (Anacker and Hen, 2017).

## Impairment of Adult Hippocampal Neurogenesis and Affective Disorders

A paradigm-shift insight is that impairment of AHN in the ventral hippocampus (VH) may affect the normal physiology of DG, contributing to affective disorders (Anacker and Hen, 2017). It has been proposed that pattern separation and cognitive flexibility impairments may underlie human affective disorders, including depression, anxiety and post-traumatic stress disorder (PTSD) (Anacker and Hen, 2017). Pattern separation is a neural computational process by which the DG circuits allow discrimination of highly similar events, contexts and environments (Sahay et al., 2011a). In behavioral level, individuals with normal pattern separation function remember highly similar events, places and even emotional contexts (Sahay et al., 2011a; Nakashiba et al., 2012; Niibori et al., 2012). Experimental ablation of AHN impairs pattern separation in rodents, while the opposite occurs by enhancing it (Clelland et al., 2009; Sahay et al., 2011b).

Cognitive flexibility is the ability to adapt to a new situation when a previous paradigm is changed, or to a changing environment in general (Burghardt et al., 2012; Park et al., 2015). It is normally attributed to the prefrontal cortex, but strong scientific evidence suggests that DG also contribute to this cognitive function (Monk et al., 2002; Takahashi et al., 2008). Reversal learning experiments have demonstrated that AHN is important for cognitive flexibility in rodents (Burghardt et al., 2012). A fundamental correlate of cognitive flexibility is the avoidance of interference of novel memories on the ones previously stored (Dupret et al., 2008; Garthe et al., 2009). AHN is supposed to be pivotal to this process (Anacker and Hen, 2017). For example, mice with experimentally impaired AHN have more difficult to find a Morris water maze platform whose hidden position was previously changed (Dupret et al., 2008; Garthe et al., 2009). It has been suggested that new-born neurons in the DG weaken the memory of the previous position of the platform, facilitating new associations and thus contributing to cognitive flexibility by decreasing proactive interference in mice (Epp et al., 2016).

It has been recently suggested that a reduction in the AHN caused by chronic stress, mainly in the VH, might impair pattern separation and cognitive flexibility, contributing to affective disorders, including depression, anxiety and PTSD, even in humans (Anacker and Hen, 2017; Toda et al., 2019). Reduced hippocampal volume and low numbers of hippocampal neural progenitors have been reported in depressed humans (Campbell et al., 2004; Lucassen et al., 2010). In addition, some anti-depressants provide therapeutic actions by enhancing AHN in mice (Santarelli et al., 2003) and humans (Boldrini et al., 2009), although some reports do not confirm these findings in further mouse experiments (Huang et al., 2008).

Affective disorders are likely hyperactivation or hypoactivation of physiological neural circuits with downstream neurochemical changes. Projections from VH to medial prefrontal cortex mediate anxiety, context aversion and behavior inhibition in mice (Adhikari et al., 2010; Padilla-Coreano et al., 2016), while bilateral projections to amygdala contribute to fear processing and memories with emotional content even in humans (Richardson et al., 2004).

Impaired adult neurogenesis following chronic stress might dysregulate such neural pathways, contributing to anxiety and depression (Anacker and Hen, 2017; Toda et al., 2019). Several stressors reduce adult neurogenesis in the VH, which contribute to depression and anxiety-like symptoms in both rodents (Hill et al., 2015; Anacker et al., 2018) and primates (Pereira et al., 2011). In this situation, stress might dysregulate the normal physiology of DG by decreasing adult neurogenesis in the VH, which regulates stress pathways by inhibiting the DG itself and its outputs to other CNS regions, including hypothalamus (Mayer et al., 2006; Snyder et al., 2011; Anacker et al., 2018).

Rodent studies suggest that diminished DG inhibition by adult-born neurons might result in hyperactivation of hypothalamus–pituitary–adrenal (HPA) axis resulting in increased levels of glucocorticoid release in a vicious cycle detrimental to AHN (Anacker, 2014). A controlled level of AHN is an important factor for stress resilience as new hippocampal neurons afford ER by inhibiting the ventral DG in mice (Anacker et al., 2018). These data raise the possibility that modulation of AHN becomes a promising interventional therapy for affective disorders (Shors et al., 2014, 2018; Anacker and Hen, 2017; Millon and Shors, 2019; Lavadera et al., 2020). This is a reasonable possibility considering that pattern separation and cognitive flexibility may be impaired in affective disorders and they depend on AHN even in humans (Déry et al., 2013; Keith et al., 2015; Anacker and Hen, 2017; Toda et al., 2019).

Impaired pattern separation and cognitive flexibility have been reported in depressed humans (Déry et al., 2013). Cognitive flexibility impairment has also been reported in patients with PTSD (Keith et al., 2015) and obsessive-compulsive disorders (Chamberlain et al., 2007). Interestingly, it has been recently reported that memory suppression is fundamental for resilience after traumatic events and that deficits in brain circuits important for memory suppression may underlie PTSD pathophysiology (Mary et al., 2020). It might be possible that deficits in AHN may contribute to this phenomenon. Impaired AHN might underlie the pathophysiology of several affective disorders in humans (Anacker and Hen, 2017; Toda et al., 2019).

Although this paper discusses the etiology of affective disorders, such as de-depression and anxiety, as related to impairment of memory-associated DG functions like pattern separation and cognitive flexibility, anxiety behavior and other affective disorders might be related to other factors, including malfunction of reward processing and innate components (Gray and McNaughton, 2000). This has been investigated in the context of obsessive-compulsive disorder (OCD) in which a lack of reward may contribute to an anxious behavior (Cohen et al., 2003; Alves-Pinto et al., 2019; Moreira et al., 2020). Further studies should investigate a possible contribution of AHN impairment to malfunction of reward system and its relation to anxiety and other affective disorders.

## An Evolutive Reason to Explain Why Aerobic Exercise and Enriched Environment Are Beneficial for Brain Cognition and Psychic Well-Being

As we mentioned before, aerobic exercise is a powerful inducer of AHN in rodents (van Praag et al., 1999) and humans (Shors et al., 2014; Anacker and Hen, 2017; Millon and Shors, 2019). An important teleological question to be raised is why are we so dependent of aerobic exercise for body fitness and how is this linked to adult neurogenesis and psychic well-being? The answer to this question may has its roots in our evolutive history (Raichlen and Polk, 2013; Raichlen and Alexander, 2017; Wallace et al., 2018). Our last common ancestor with apes was some kind of arboreal quadrupedal ape that lived in African forests between 8 and 5 million years ago (Wallace et al., 2018). Current apes and the various hominids of the genus Homo descend from this common ancestor, which were adapted to tree climbing (Wallace et al., 2018). Early hominids, such as *Homo erectus*, evolved to walk in a bipedal posture and were hunter-gatherers for million years (Marlowe, 2005). They did not have hunting tools and used to collect food and practicing persistence hunting, a technique in which the animals were chased until they were exhausted allowing their capture (Liebenberg, 2006).

During the endurance hunting, early hominids practiced intense aerobic activity walking or running for up to 15 km per day to capture animals. Over million years they progressively developed an important sense of cooperation to accomplish successful obtention of fresh food (Hill, 2002; Raichlen and Polk, 2013; Raichlen and Alexander, 2017; Wallace et al., 2018). It has been hypothesized that our hunter-gatherer ancestors gradually developed complex cognitive skills from learning deductive reasoning during search for animal trail, which were then chosen by natural selection, contributing to the increase of brain volume and complexity of canonical circuits over the course of evolution (Hill, 2002; Henrich, 2016). It follows that it is possible that logical-deductive reasoning and other important cognitive skills emerged from these hunting activities of our hunter-gatherer ancestors (Liebenberg, 1990; Wallace et al., 2018).

Based on the evolutive history of primitive humans as hunter-gatherers it is reasonable to suppose that there was a co-evolution of our brain capabilities with aerobic exercise, considering that for about 2 million years our ancestors lived in close contact with nature performing intense walking and running, which were extremely needed for their survival. Only about 10 thousand years ago farming was invented by humans, which allowed settlement of communities in fixed living places with progressive abandonment of their nomadic lives as hunter-gatherers. This was an important step for the origin of modern societies, but a great change in the long-lasting human habits of walking and running during hunting and vegetable gathering.

Scientific evidences suggest that there was an evolutionary relationship between aerobic physical activity and brain evolution (Raichlen and Polk, 2013; Raichlen and Alexander, 2017). For example, a considerable increase in brain size occurred during the evolution of Homo erectus (Holloway, 2008; Sherwood et al., 2008; Raichlen and Polk, 2013). In about 2 million years of Homo erectus existence, brain size increased 600–700 to 1,200 cm^3^ (Raichlen and Polk, 2013; Raichlen and Alexander, 2017; Wallace et al., 2018). The phenomenon was even more pronounced in species that emerged after Homo erectus in which brain volume reached 1,170–1,740 cm^3^ in Homo neanderthalensis to 1,100–1,900 cm^3^ in Home sapiens. This was coincident with adaptations for endurance, higher use of aerobical activity and hunter-gatherer lifestyle (Raichlen and Polk, 2013; Raichlen and Alexander, 2017; Wallace et al., 2018).

Another evidence that increased aerobic activity by hominids contributed to human brain evolution and increased cognitive capacities comes from comparative evolution studies on the variables related to endurance capacities and their relation to brain evolution (Bramble and Lieberman, 2004; Raichlen and Polk, 2013). The variables hindlimb length and semicircular canal dimensions were investigated in great apes, humans and hominin taxa in an attempt to perform correlations between the contribution of those variables for higher endurance performance and brain size (Bramble and Lieberman, 2004). The results have shown that in extant humans, hindlimbs were adapted to be longer than any similarly sized quadrupeds, which contributed to high endurance running speed, reducing energy consumption for longer aerobic activity like during running or rapid walking (Bramble and Lieberman, 2004).

The semicircular canal system, which is important for specific ocular, head and body movements allowing stability, was also shaped for allowing better performance during endurance running and other energy-consuming aerobic activity (Spoor et al., 1994; Lieberman et al., 2007). For example, larger semicircular canals were present in animals performing faster and agile locomotor behaviors causing higher angular acceleration of the head (Spoor et al., 1994). This was the case for humans and Homo erectus, which presented increased anterior and posterior semicircular canal radii compared with earlier hominins and great apes. When correlation analyses were performed between these two variables and encephalization quotients (EQ) in great apes, humans and early hominids with body mass correction, a positive correlation was found (EQ and hindlimb length, *r* = 0.93; anterior (*r* = 0.77) and posterior semicircular canal radii (*r* = 0.66). These data suggest a correlation between traits related to endurance activity and brain evolution.

An important question to address is how millions of years of hunter-gatherer life and daily endurance activity influenced brain developing and cognition? It has been proposed that intense and continuous aerobic activity influenced brain cognition by shaping systems of growth factors (Raichlen and Polk, 2013). There is also evidence that selective pressure on neurotrophin and growth factor signaling may have considerable impact on brain evolution (Raichlen and Gordon, 2011). Studies in rodents, primates and humans suggest that levels of neurotrophin and other growth factors correlate with brain size (Raichlen and Gordon, 2011; Raichlen and Polk, 2013).

Voluntary and forced running up regulates levels of brain-derived neurotrophic factors (BDNF) in rodent and human brains, which contributes to neuronal survival and increased adult neurogenesis (Neeper et al., 1995; Zoladz et al., 2008; Horowitz et al., 2020). Long physical training up regulates levels of BDNF, insulin-like growth factor-1 (IGF-1) and VEGF in humans even at rest, which suggests that continuous physical activity up regulates neuroplasticity (Neeper et al., 1995; Gustafsson et al., 2001; Kraus et al., 2004; Zoladz et al., 2008; Raichlen and Polk, 2013). Physical aerobic activity first upregulates levels of growth factors in the periphery regulating metabolism and vascular activity, which is important for physical activity (Kraus et al., 2004). Second, growth factors may get into the brain contributing to neurogenesis and cognition which is important for exploratory activity using locomotion (Klein et al., 2011).

Such events might be occurred over the course of hominid evolution. Shift to hunter-gatherer life style with continuous aerobic physical activity from Homo erectus and beyond, likely influenced systems of growth factors including IGF-1 and VEGF first in the body periphery for metabolic purpose and later inside the brain contributing to increased brain cognition (Raichlen and Polk, 2013). This is supported by intra and interspecific *in vivo* experiments suggesting that levels of brain growth factors and brain size in different mammalian species correlates with aerobic physical activity performance (Johnson and Mitchell, 2003; Chappell et al., 2007; Raichlen and Gordon, 2011; Raichlen and Polk, 2013). In addition, experiments using artificial selection in rodents have shown that evolutive selection of variables associated with athletic ability and aerobic activity performance have profound effects on brain size and cognition over generations (Swallow et al., 1998; Bye et al., 2008; Wikgren et al., 2012; Kolb et al., 2013). This in agreement with the hypothesis that over the evolution of human brain selective pressure on non-cognitive variables like aerobic physical activity has profound influences on brain size and cognition (Raichlen and Polk, 2013; Hill and Polk, 2019).

Considering that continuous aerobic exercise shaped brain evolution and developing, that is the likely explanation for the fact that running increases adult hippocampal neurogenesis (van Praag et al., 1999). It is possible that over the course of human evolution there was a kind of “neural symbiosis” between brain, physical activity and nature (natural landscapes), which might explain why aerobic exercise and enriched environment are so important for adult neurogenesis and cognition. This is supported by the fact that maturation of CNS circuits is dependent on sensory and motor stimuli (Chow et al., 1957; Narducci et al., 2018). Further experiments are needed to confirm this hypothesis. One such an experiment would be to investigate the effects of outdoor (parks with beautiful landscapes and lots of nice sensory stimuli) and indoor (ordinary fitness centers) aerobic physical activities in the mood of patients with depression and anxiety. In addition, monitoring of hippocampal volume would be interesting as it has been shown that this quantitative variable is influenced by physical activity and it is decreased in chronic depression and anxiety (Gilbertson et al., 2002; Campbell et al., 2004; Anacker and Hen, 2017).

Some authors suggested that the human species is not yet completely adapted to the environment of modern societies, including modern cities (Wallace et al., 2018). This can be an outcome of our past evolutive history as hunter-gatherers living in natural environments (Raichlen and Polk, 2013; Raichlen and Alexander, 2017).

A recent study confirmed the benefit of aerobic exercise for both rodent and human brain (Horowitz et al., 2020). Authors have shown that blood factors transfer from young and old runner mice to sedentary mice improves hippocampal neurogenesis and cognitive performance in behavioral tests. They also identified the enzyme released by the liver glycosylphosphatidylinositol (GPI)–specific phospholipase D1 (Gpld1) as an important factor mediating the effects on brain cognition and reported that this enzyme is up regulated into the blood of elderly humans who practice regular physical activity, but not in sedentary elderlies. This study established a strong link between muscular activity and brain cognition in agreement with a previous paper reporting a similar role for the myokine irisin in an experimental model of Alzheimer disease in mice (Lourenco et al., 2019).

Further anthropological and historical studies should investigate whether in the past, before massive human migration from the countryside to cities and industrial revolution, affective disorders like depression and anxiety were less common. Nevertheless, it is very likely that the link between AHN and physical activity was shaped during human evolution as a byproduct of the intense physical activity of our hunter-gatherer ancestors. It follows that we did not evolve to be physically inactive (Wallace et al., 2018).

## The Human Correlate of an Enriched Environment: Running in a Natural Landscape

Like aerobic exercise, enrichment environment is a powerful inducer of hippocampal neurogenesis (Kempermann et al., 1997; Kempermann, 2019). Rodents living in cages with enriched environment allowing higher stimulation of their mystacial vibrissae have more hippocampal new-born cells than animals living in plain cages (Kempermann et al., 1997). In addition, other aspects of an enriched environment for rodents include more room to roam, places to hide, spatial complexity, navigational demand (Kempermann et al., 1997; Kempermann, 2019).

Rodents are not visual animals and obtain most of the information from environment using their somatosensory system. Each mystacial vibrissae present in their snout has a modular representation onto the somatosensory cortex (Woolsey and Van Der Loos, 1970). Despite the importance of somatosensory cortex in primates, including humans, most of the sensory information is obtained by the visual system (Knudsen, 2020).

It has been established that motor and sensory stimuli in an enriched environment have powerful effects on AHN in rodents (Kempermann et al., 1997; Kempermann, 2019). Here, we propose that beyond motor stimuli, sensory stimuli have an important contribution to human hippocampal neurogenesis and psychic well-being. It is very common that humans performing physical activities like walking, jogging or running in outdoor environments with beautiful landscapes, bird sounds, wind, nice flower smells report a tremendous feeling of pleasure and well-being. Like for motor activity, the beneficial effect of sensory stimulation for adult neurogenesis and cognition is likely an evolutive consequence of the bucolic lives of our hunter-gatherer ancestors who lived for million years in an environment with natural landscapes (Wallace et al., 2018).

I would like to propose here that pleasant sensory stimuli contribute to psychological well-being through neural pathways connecting sensory cortices to neurogenic areas in DG and then to reward centers. There is a neuroanatomical basis for this hypothesis. Several sensory pathways project to entorhinal cortex, which send axons to hippocampal DG in several species, including rodents, primates and humans (Knierim et al., 2013; Luna et al., 2019). From the ventral DG, there are axonal projections to CA3 and then to reward centers, including nucleus accumbens (NA) in the ventral striatum of mice (Britt et al., 2012; Bagot et al., 2015). The release of dopamine and serotonin in the reward centers contributes to hedonic feelings, including pleasure and happiness in humans (Worley, 2017; Loonen and Ivanova, 2019). There is a complex reciprocal interaction between new-born cells of the DG and neurons located in the lateral or medial part of the entorhinal cortex in mice (Luna et al., 2019).

**FIGURE 1:**
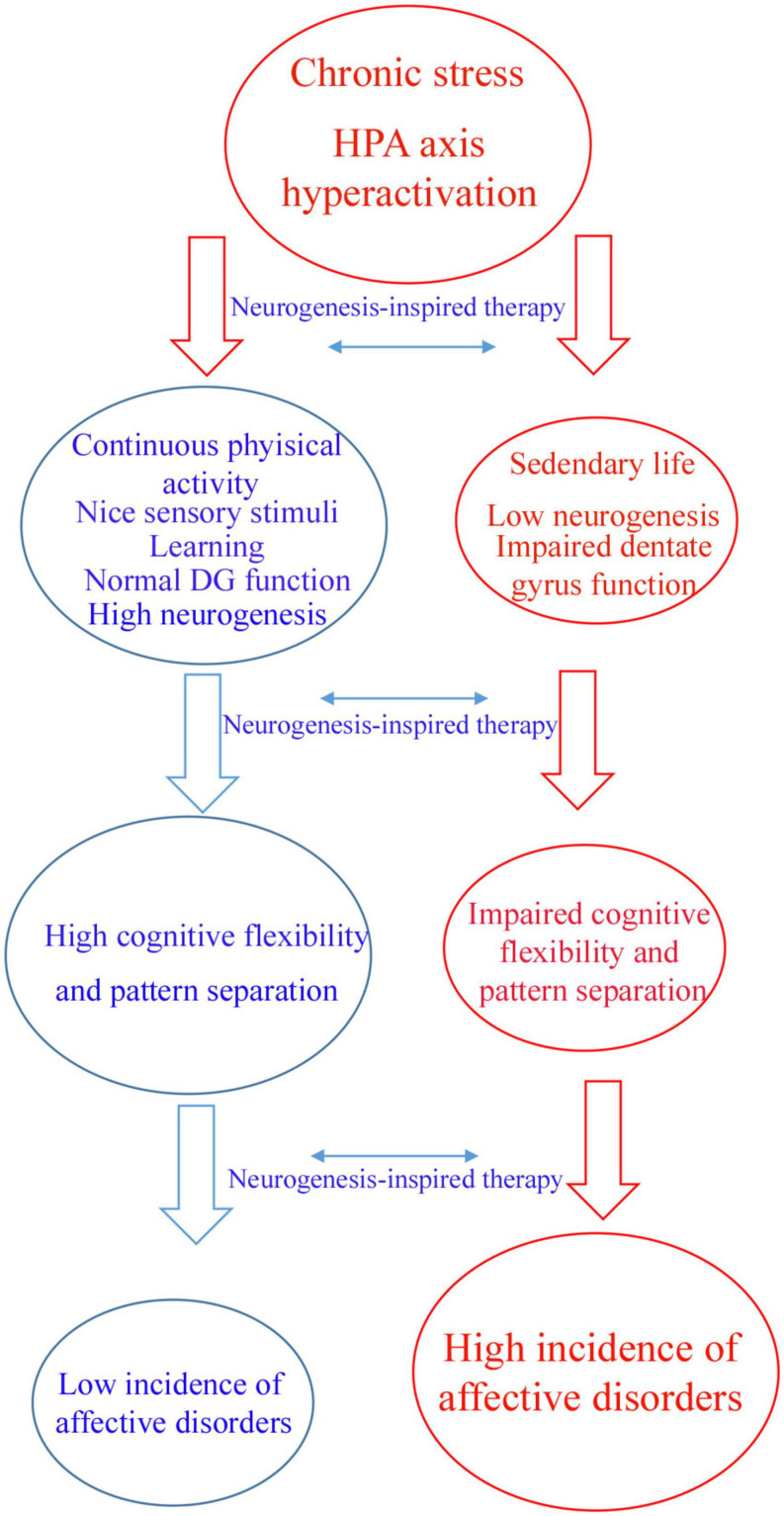
Flowchart illustrating a hypothetic mechanism by which neurogenic-enhancer activities may protect the brain against affective disorders. Chronic stress can induce hyperactivity of hypothalamus–pituitary–adrenal (HPA) axis which is a main factor contributing to affective disorders. Individuals who practice continuous aerobic physical active, meditation, learning and other neurogenic enhancer activities maintain physiological levels of adult hippocampal neurogenesis, patter separation and cognitive flexibility avoiding affective disorders like depression and anxiety (blue arm). According to this hypothesis, sedentary individuals are more affected by chronic stress presenting low levels of adult hippocampal neurogenesis and impaired dentate gyrus function, including pattern separation and cognitive flexibility (red arm). The latter group of individuals are more affected by affective disorders like depression and anxiety. Neurogenesis-inspired therapies, like MAP-training might reverse symptoms of affective disorders in susceptible patients.

Therefore, according to the proposed hypothesis, motor and sensory stimuli have profound effects on AHN, contributing to psychic well-being (Figure 1). This was likely shaped by our evolutive history. In addition to the tremendous benefit for body conditioning, I would like to raise the hypothesis that aerobic exercise preferentially performed in an outdoor environment with beautiful landscapes and a plenty of nice sensory stimuli are beneficial for the mind (Figure 1). There are evidences that aerobic exercise during dancing and meditation decreases symptoms of affective disorders (Shors et al., 2014, 2018; Anacker and Hen, 2017; Millon and Shors, 2019).

Cognitive therapies must now include neurogenesis- inspired interventions aiming at increasing AHN in order to activate reward centers, which are inhibited in depression by axons from lateral habenula according to experiments in mice (Yang et al., 2018). In addition, it has been shown that there are specific neural pathways from the VH to prefrontal cortex that contribute to anxiety in mouse models (Felix-Ortiz et al., 2013; Padilla-Coreano et al., 2016). New-born DG cells contribute to stress resilience by inhibiting mature granule cells in the ventral DG of mice (Anacker et al., 2018).

## Neurogenesis-Inspired Therapies for Affective Disorders

As already discussed, experimental evidence in both human and rodents, points to a clear link between functional impairment of adult neurogenesis in the DG and deficits in pattern separation and/or cognitive flexibility. Some studies suggest that this may occur during normal aging and as part of the pathophysiology of affective disorders (Anacker and Hen, 2017). A promising approach in modern psychology is the development neurogenesis-inspired therapies in humans (Cole et al., 2012; Mishra et al., 2016; Anacker and Hen, 2017). These cognitive therapies aim at enhancing AHN use techniques including aerobic exercise/meditation (Shors et al., 2014; Millon and Shors, 2019) and brain-training games engaging hippocampal function (Cole et al., 2012; Mishra et al., 2016). This is illustrated by the *physical and mental training* (MAP-training), an approach developed by Tracey Shors and colleagues at the department of psychology of Rutgers University (New Jersey, United States). This approach relies on meditation techniques such “mindfulness,” and continuous aerobic exercise, for the treatment of affective disorders, such as depression, anxiety and PTSD (Shors et al., 2014, 2018; Millon and Shors, 2019).

MAP-training was initially applied in a pilot study as a therapy for homeless women with history of physical and sexual abuses, domestic violence, extreme poverty, addiction, and depression (Shors et al., 2014). In short, the MAP-training intervention protocol comprises activities inspired on results obtained from animal laboratory experiments showing that aerobic exercise and learning tasks increase the number of new neurons in the hippocampal DG of rodents (van Praag et al., 1999; Curlik and Shors, 2011) and contribute to their survival (Gould et al., 1999; Curlik and Shors, 2011; Shors et al., 2012), respectively.

In MAP-training intervention, patients were asked to perform focused attention meditation (“mindfulness”) for 20 min in a sit position, followed by 10 min of walking meditation. Finally, patients were asked to perform aerobic exercise for 30 min in a funny way involving dancing activities, including Zumba and Jazzercise. Sessions were performed twice a week for 8 weeks (Shors et al., 2014). According to the authors, the homeless women attending the study presented a significant improvement in the maximum rate of oxygen consumption and less symptoms of anxiety and depression. Homeless women not attending the study did not present such an improvement in their conditions. In a follow up study, the same group reported that MAP-Training reduced ruminative thoughts and post-traumatic feelings, while increased measures of self-worth in women victim of sexual violence (Shors et al., 2018). A similar result was recently reported for medical students attending MAP-training for 8 weeks who presented less ruminative thoughts, stress symptoms and better quality of life (Lavadera et al., 2020).

The neurogenesis-inspired therapies, such as MAP-training, are very promising, but they do not prove that improvement of symptoms of patients with depression and anxiety are due to enhanced AHN. Innovative technology to monitor adult neurogenesis in the human brain are needed and they are not available at the moment.

## Conclusion and Future Perspectives

Future studies should confirm that normal DG physiology is impaired due to impaired adult neurogenesis in humans and their contribution to the pathophysiology of affective disorders. Therapies aiming at enhancing AHN may be feasible in the future to treat depression, anxiety and other affective disorders (Anacker and Hen, 2017). Physical exercise is a powerful inducer of AHN in rodents (van Praag et al., 1999; Vivar et al., 2016) and has been already tested in humans with promising results (Shors et al., 2014, 2018; Millon and Shors, 2019). Brain-training games for improving cognition have also been tested to target hippocampal functions in humans with positive outcomes (Cole et al., 2012; Mishra et al., 2016). Future pharmacological and non-pharmacological therapies should be developed to enhance AHN in order improve pattern separation and cognitive flexibility, helping humans with affective disorders. To confirm that these neurogenesis-inspired therapies really are effective, measurement of the hippocampal volume and/or cerebral blood flow could be performed to indirectly infer changes in AHN (Gilbertson et al., 2002; Santarelli et al., 2003). In addition, non-invasive imaging technology are necessary to monitor adult hippocampal neurogenesis upon neurogenesis-inspired therapies. For example, proton magnetic resonance spectroscopy can be used to detect a specific biomarker of hippocampal progenitor cells that give rise to new granule cells in humans (Manganas et al., 2007).

## Author Contributions

The author confirms being the sole contributor of this work and has approved it for publication.

## Conflict of Interest

The author declares that the research was conducted in the absence of any commercial or financial relationships that could be construed as a potential conflict of interest.
